# Change in corneal endothelial cell density after phacoemulsification in patients with type II diabetes mellitus

**DOI:** 10.12669/pjms.35.5.596

**Published:** 2019

**Authors:** Muhammad Khalid, Muhammad Kashif Hanif, Qamar ul Islam, Muhammad Asim Mehboob

**Affiliations:** 1 Dr. Muhammad Khalid, MBBS. Department of Ophthalmology, PNS SHIFA, Karachi, Pakistan; 2 Dr. Muhammad Kashif Hanif, FCPS. Department of Ophthalmology, PNS SHIFA, Karachi, Pakistan; 3 Dr. Qamar ul Islam, FCPS. Department of Ophthalmology, PNS SHIFA, Karachi, Pakistan; 4 Dr. Muhammad Asim Mehboob, FCPS. Department of Ophthalmology, PNS SHIFA, Karachi, Pakistan

**Keywords:** Phacoemulsification, Corneal Endothelial Cell Density, Diabetes Mellitus

## Abstract

**Objective:**

To compare the mean change in Corneal Endothelial cell Density (CED), from baseline (pre-operative value), two months after phacoemulsification cataract surgery between type II diabetic patients and non-diabetic patients.

**Methods:**

This prospective stratified controlled study was conducted at PNS Shifa Hospital, Karachi. 80 eyes of 72 type II diabetic patients and 80 eyes of 77 non diabetic controls, having Nuclear Opalescence (NO) grades 2 and 3 on slit lamp examination underwent phacoemulsification surgery. CED was measured in cells/mm^2^, of concerned eye of each subject preoperatively and 2 months post operatively using specular microscope. The difference in mean CED change between the two groups after surgery was analyzed.

**Results:**

Mean age of study population was 61.41± 6.76 years. Out of study population, 92 (57.5%) were males and 68 (42.5%) were females. There was a statistically significant difference between both groups in terms of mean post-operative CED, mean change in CED and mean frequency change in CED (p <0.05). There was no statistically significant difference between both groups in age, gender, laterality of eyes and mean pre-operative CED, (p >0.05). Difference of pre-operative CED from post-operative CED in each group was statistically significant.

**Conclusion:**

There is a significant difference between diabetic population and normal population in terms of corneal endothelial loss after uneventful phacoemulsification cataract surgery.

## INTRODUCTION

Cataract is the leading cause of treatable blindness worldwide.^1^ Phacoemulsification with intraocular lens (IOL) implantation is the most common surgical procedure performed for the treatment of cataract, with benefits of causing less surgically induced astigmatism and early and better visual rehabilitation.^2^,^3^ Despite all the potential benefits of phacoemulsification, corneal endothelial cell loss remains one of the major concerns especially in patients with already compromised corneal endothelium.

Studies have showed that corneal endothelium of diabetic patients is more prone to damage after cataract surgery.^2^,^4^ The diabetic cornea suffers from cellular dysfunction and dysfunctional repair mechanisms leading to decreased corneal endothelial cell density (CED).^5^ This makes cornea vulnerable to surgical insults as corneal endothelium lacks the ability to regenerate. Moreover, the diabetic patients opting for cataract surgery are usually old aged and have less CED since the number of endothelial cells decreases with age. As a result, it is recommended to evaluate the corneal endothelium routinely prior to phacoemulsification, particularly in diabetic patients.^4^

Specular microscopy is a non invasive photographic technique that analyzes the size, shape and population of the endothelial cells. This technique can be effectively utilized to assess CED in patients undergoing phacoemulsification and gives an estimate of loss of endothelial cells postoperatively. Hugod M et al. in their study on 60 patients reported that mean frequency decrease in CED after phacoemulsification in diabetic patients was 6.2%, compared to 1.4% in normal controls.^2^ No study has been conducted in Pakistan to compare the loss of CED in diabetic patients with normal population. This study will provide an insight into the pattern of CED changes after phacoemulsification in our diabetic population in comparison to normal controls. Estimation of CED changes will help the surgeon in doing appropriate management per operatively in diabetic patients to avoid significant and permanent damage to corneal endothelium.

## METHODS

After approval by the Ethical Review Committee PNS SHIFA, informed consent was taken from the subjects prior to inclusion in the study. The study started in Nov 2016 and ended in Feb 2018. Patients from either gender, with cataract density of NO 2 and NO 3 according to Lens Opacity Classification System III and Axial Length (AL) between 23 and 25mm were included. Patients with history of corneal diseases, dystrophies, previous ocular surgery, previous topical ocular medications used for more than six months, trauma, prolonged surgery, complicated surgery and pre-operative CED of <2000 cells/mm^2^ were excluded. After fulfilling the inclusion criteria, patients were assigned into two groups; Group-1 comprised diabetic cataract patients and Group-2 involved non diabetic cataract patients. The Group-1 patients were diagnosed cases of Type-2 diabetes mellitus based on their medical record and were on oral hypoglycaemics with good glycaemic control and no diabetic retinopathy. Prior fasting blood sugar levels, serum HbA1C and random blood sugar levels (on operation day) were obtained in Group-1 patients to evaluate their glycaemic status. All patients underwent detailed anterior and posterior segment examination, and planned for cataract surgery. CED in all patients was evaluated preoperatively by Topcon SP 3000P Specular Microscope (Topcon Corporation, Tokyo, Japan). AL was measured using Intraocular Lens (IOL) Master. All patients underwent phacoemulsification cataract surgery by a single experienced surgeon using same phaco machine (Oertli, Faros, Switzerland). Patients were reviewed postoperatively to rule out any complication and CED was recorded after two months. The data was recorded in a comprehensive proforma for each patient. Statistical Package for Social Sciences (SPSS 22.0) for windows was used for statistical analysis. Descriptive statistics i.e. mean ± standard deviation for quantitative values (age, Pre-operative CED, Post-operative CED, mean change in CED, mean frequency change in CED) and frequencies along with percentages for qualitative variables (gender, laterality) were used to describe the data. Shapiro Wilk’s test was used to check normality of data. Post normality testing, Chi square test was used to compare qualitative variables, and independent t test to compare quantitative variables between two groups. Moreover, Paired ‘t’ test was used to compare postoperative value from pre-operative value within each group. P value < 0.05 was considered statistically significant.

## RESULTS

A total of 160 eyes of 149 patients were included in the study. Mean age of study population was 61.41± 6.76 years. Out of study population, 92 (57.5%) were males and 68 (42.5%) were females. Out of operated eyes, 80 (50%) were right, and 80 (50%) were left eyes. Mean age, mean pre-operative CED, mean post-operative CED, mean change in CED and mean frequency change in CED of study population is given in Table-I. There was no statistically significant difference between both groups in terms of age, gender, laterality of eyes and mean pre-operated CED (p >0.05). However, mean post-operative CED, mean change in CED and mean frequency change in CED showed a statistically significant difference (p <0.05). The comparison of pre-operative CED from post-operative CED within each group is given in Table-II. Difference of pre-operative CED from post-operative CED in both groups was statistically significant.

**Table I:** Group Wise Demographic Data (n=160).

Characteristic	Study Population (n=160)	Group 1 (n=80)	Group 2 (n=80)	p Value (Between groups)
Age (Years)Mean ± SD	61.41± 6.76	61.58±7.65	61.24± 5.78	0.753**
Gender (Male/Female)	92/68	47 / 33	45 / 35	0.749*
Laterality (Right/Left)	80/80	36/44	44/36	0.206*
Pre-Op CED (Cells/mm^2^) Mean ± SD	2648.88±345.91	2639.89±331.99	2657.87±361.16	0.743**
Post-Op CED (Cells/mm^2^) Mean ± SD	2325.17±444.05	2250.37±426.68	2399.97±451.08	0.033**
Mean Change in CED (Cells/mm^2^) Mean ± SD	323.71±283.08	389.51±285.37	257.90±266.61	0.003**
Mean Frequency Change in CED (%) Mean ± SD	12.37±10.77	14.88±10.79	9.86±10.20	0.003**

* Chi Square test

** Independent t test

**Table II:** Pre-op and Post-op comparison of two groups.

Variable	Group 1 (n=80)	Group 2 (n=80)
CED (Cells/mm^2^)	Mean±SD	
Pre-operative	2639.89±331.99	2657.87±361.16
Post-operative	2250.37±426.68	2399.97±451.08
p Value*	<0.001	<0.001

* Paired t test

## DISCUSSION

Phacoemulsification with IOL implantation is a swift and delicate surgical procedure that involves small incision site. It is the most commonly performed surgery worldwide for the treatment of cataract and is considered very safe, though surgical hand, technique, experience of surgeon and the patient and other factors influence final outcome. Since the procedure involves use of high ultrasound energy phaco tip and the other instruments in a very small space of anterior chamber, the corneal contact of those instruments is inescapable. Such a contact with cornea mainly damages corneal endothelium layer which consists of a layer of hexagonal cells with very important functions that maintain corneal integrity and clarity through their pump mechanism. Corneal endothelial cells cannot regenerate, their loss (CED loss) brings about compromise of the dehydration pump system of cornea leading to hydration and loss of clarity (compromised cornea). 6 Hence, CED loss remains one of the major concerns for surgeons especially in older patients with already compromised corneal endothelium.

The state of corneal endothelium in diabetic patients has been investigated by many studies with the use of specular microscope and most of them agree that the corneal endothelium of diabetic patients has morphologically abnormal features as compared to non diabetic controls that include loss of hexagonality, decreased CED, greater coefficient of variance and greater Central Corneal Thickness (CCT).^5^,^7^,^8^ The overall effect is instability and vulnerability to even minor surgical trauma. Diabetics with glycaemic control with medications also have shown these features. This implies that diabetes mellitus is a potential non modifiable risk factor of CED loss after phacoemulsification. The other risk factors include age, short AL and anterior chamber depth, cataract density, corneal incision size, phacoemulsification time, mean ultrasound power, lens fragment and IOL contact during insertion, viscoelastic substance used etc.^6^,^9^,^10^ To minimize the effects of such factor on our study outcome, we selected same age group patients (40-60 years), with particular AL (23 mm – 25 mm) and cataract density (NO 2 and NO 3) and limited phacoemulsification time (<40 sec); surgical factors were minimized by similar surgical technique by same surgeon with same phaco machine in all cases.

Studies are underway to observe CED loss in diabetic patients in comparison with non diabetic controls after small incision cataract surgeries. Many studies have already indicated that diabetic corneas undergo more morphological as well functional damage during surgery and post operative edema resolution is delayed. Our results are consistent with them. Hugod M et al. reported a significant CED loss and a delayed recovery form corneal edema in diabetic patients.^2^ They included 30 patients with type 2 diabetes and 30 controls without diabetes and observed mean CED loss of 6.2% in diabetic group as compared to 1.4% in control group. This study was comprehensive but still had limitations of neglecting influence of NO and phaco time on final outcome. Similar conclusion was given by Morikubo et al. and recommended adoption of advanced small incision techniques in diabetic patients to ensure minimum surgical trauma.^11^ Our study results showed mean CED loss of 14.88% (±10.79) in diabetic group, as compared to 9.86% (±10.20) in control group with a p value of 0.003 (<0.05). In our study, we tried to address the effects of many risk factors which could influence final results. The results endorse previous studies and the study is first of its kind in Pakistan. Further such studies with more parameter and with large sample sizes are recommended.

### Authors Contribution

**MK:** conceived, designed, wrote & edited manuscript.

**MKH & QUI:** did editing & final approve of manuscript.

**MAM:** did statistical analysis & review of manuscript.

## References

[1] Mohammadi SF, Hashemi H, Mazouri A, Rahman AN, Ashrafi E, Mehrjardi HZ (2015). Outcomes of cataract surgery at a referral center. J Ophthalmic Vis Res.

[2] Hugod M, Paulsen AS, Norregaard JC, Nicolini J, Larsen AB, Thulesen J (2011). Corneal endothelial cell changes associated with cataract surgery in patients with type 2 diabetes. Cornea.

[3] Zhang JY, Feng YF, Cai JQ (2013). Phacoemulsification versus manual small-incision cataract surgery for age-related cataract:meta-analysis of randomized controlled trials. Clin Experiment Ophthalmol.

[4] Yang R, Sha X, Zeng M, Tan Y, Zheng Y, Fan F (2011). The influence of phacoemulsification on corneal endothelial cells at varying blood glucose levels. Eye Sci.

[5] Sudhir RR, Raman R, Sharma T (2012). Changes in corneal endothelial cell density and morphology in patients with type 2 diabetes mellitus:a population-based study, Sankara Nethralaya Diabetic Retinopathy and Molecular Genetics Study (SN-DREAMS, Report 23). Cornea.

[6] Waring GO III, Bourne WM, Edelhauser HF, Kenyon KR (1982). The corneal endothelium. Normal and pathologic structure and function. Ophthalmol.

[7] Agamy AE, Alsubaie S (2017). Corneal endothelium and central corneal thickness changes in type 2 diabetes mellitus. Clin Ophthalmol.

[8] Galgauskas S, Laurinaviciute G, Norvydaite D, Stech S, Asoklis R (2015). Changes in Choroidal Thickness and Corneal Parameters in Diabetic Eyes. Eur J Ophthalmol.

[9] Storr-Paulsen A, Norregaard JC, Ahmed S, Storr-Paulsen T, Pedersen TH (2008). Endothelial cell damage after cataract surgery:divide-and-conquer versus phaco-chop technique. J Cataract Refract Surg.

[10] Holzer MP, Tetz MR, Auffarth GU, Welt R, Volcker HE (2001). Effect of Healon 5, 4 and other viscoelastic substances on intraocular pressure and endothelium after cataract surgery. J Cataract Refract Surg.

[11] Morikubo S, Takamura Y, Kubo E, Tsuzuki S, Akagi Y (2004). Corneal changes after small incision cataract surgery in patients with diabetes mellitus. Arch Ophthalmol.

